# Complement Potentiates Immune Sensing of HIV-1 and Early Type I Interferon Responses

**DOI:** 10.1128/mBio.02408-21

**Published:** 2021-10-12

**Authors:** Wilfried Posch, Marta Bermejo-Jambrina, Marion Steger, Christina Witting, Gabriel Diem, Paul Hörtnagl, Hubert Hackl, Cornelia Lass-Flörl, Lukas A. Huber, Teunis B. H. Geijtenbeek, Doris Wilflingseder

**Affiliations:** a Institute of Hygiene and Medical Microbiology, Medical University of Innsbruck, Innsbruck, Austria; b Department of Experimental Immunology, Amsterdam Infection and Immunity Institute, Academic Medical Center, University of Amsterdam, Amsterdam, Netherlands; c Institute of Human Genetics, Medical University of Innsbruck, Innsbruck, Austria; d Central Institute for Blood Transfusion and Immunological Department, Innsbruck, Austria; e Institute of Bioinformatics, Biocenter Innsbruck, Medical University of Innsbruck, Innsbruck, Austria; f Institute of Cell Biology, Biocenter Innsbruck, Medical University of Innsbruck, Innsbruck, Austria; University of Melbourne; Columbia University/HHMI

**Keywords:** HIV-1, dendritic cell, complement, CR4, type I IFN, antiviral immunity, cytosolic sensor, complement receptors, dendritic cells, human immunodeficiency virus

## Abstract

Complement-opsonized HIV-1 triggers efficient antiviral type I interferon (IFN) responses in dendritic cells (DCs), which play an important role in protective responses at the earliest stages in retroviral infection. In contrast, HIV-1 suppresses or escapes sensing by STING- and MAVS-associated sensors. Here, we identified a complement receptor-mediated sensing pathway, where DCs are activated in CCR5/RLR (RIG-I/MDA5)/MAVS/TBK1-dependent fashion. Increased fusion of complement-opsonized HIV-1 via complement receptor 4 and CCR5 leads to increased incoming HIV-1 RNA in the cytoplasm, sensed by a nonredundant cooperative effect of RIG-I and MDA5. Moreover, complement-opsonized HIV-1 down-modulated the MAVS-suppressive Raf-1/PLK1 pathway, thereby opening the antiviral recognition pathway via MAVS. This in turn was followed by MAVS aggregation and subsequent TBK1/IRF3/NF-κB activation in DCs exposed to complement- but not non-opsonized HIV-1. Our data strongly suggest that complement is important in the induction of efficient antiviral immune responses by preventing HIV-1 suppressive mechanisms as well as inducing specific cytosolic sensors.

## INTRODUCTION

Incoming pathogens are recognized by antigen-presenting cells such as dendritic cells (DCs), Langerhans cells (LCs), or macrophages via an array of pattern recognition receptors (PRRs), including Toll-like receptors (TLRs), C-type lectin receptors (CLRs), and, importantly, complement receptors (CRs). The significance of complement with respect to inducing innate and adaptive immunity in response to pathogens via intrinsic or receptor-activating functions was recently highlighted (1–4). DCs were illustrated to produce innate cytokines, including type I interferons (IFN-α and IFN-β), interferon-stimulated genes (ISGs), proinflammatory cytokines (interleukin 1β [IL-1β], IL-6, and IL-23), and the complement anaphylatoxin C3a, when exposed to HIV-1 surrounded by covalently linked complement C3 fragments (2, 3). Such complement-opsonized HIV-1 (HIV-C) also mediated significantly enhanced DC infection, DC maturation, and stimulation of efficient HIV-1-specific cytotoxic-T-lymphocyte (CTL) responses (2, 5). In contrast, non-opsonized HIV-1 (HIV) was illustrated to manipulate DCs to mediate only partial DC maturation and to efficiently transmit virus to target T cells via the virological synapse (6). This partial DC maturation by HIV resulted in migration and enhanced DC/T cell interactions but a lack of efficient antiviral activity (7).

The enhancement of type I IFN production mediated via HIV-C might play a major protective role at the earliest stages of infection. HIV-1 is spontaneously coated with C3 fragments in semen (8) and at mucosal sites due to a C1q-binding site in the envelope glycoprotein gp41 (9, 10). Since it binds regulators of complement activation (RCAs) and fluid phase factor H (fH), the virus is not destroyed by complement-mediated lysis (11), and most HIV-1 particles persist covered with covalently bound C3 fragments in the host. Complement-opsonized HIV-1 was found accumulated in all so-far-tested compartments of HIV-positive individuals, such as mucosa, germinal centers, and seminal fluid (8, 12, 13), and when particles are opsonized *in vitro* using seminal fluid or normal human serum (NHS), immediate C3 deposition on the viral surface is observed (2, 3, 10). Such opsonized virus particles represent better models to mimic early-stage HIV-1 pathogenesis than non-opsonized HIV-1, since in simian immunodeficiency virus (SIV)-infected rhesus macaques the protective effects of an early type I IFN activation was also emphasized (14, 15). These protective effects might be attributed to the recognition of HIV-1/SIV by complement and subsequent interactions with CRs on DC subsets. Not only SIV and HIV-C but also HIV-2 exerted improved capacity to activate antiviral innate immune pathways due to DC infection and improved recognition (2, 3, 5, 16, 17), while HIV has only poor ability to efficiently infect and activate DCs (16, 18, 19). HIV-2 infection of DCs mediated type I IFN expression via NONO binding to the capsid, thus promoting viral DNA sensing by cGAS, while HIV-1 capsid showed a lower affinity for this innate immune sensor, thereby escaping detection by DCs (20). A further mechanism to explain avoidance of HIV-1 sensing by DCs was recently described by Gringhuis et al. (21) and comprises DC-SIGN interactions. HIV-1 recognition by DC-SIGN resulted in activation of PLK1 by Raf-1, which blocked downstream regulation of MAVS and subsequent type I IFN induction (21).

Here, we investigated the role of complement in the detection of HIV-1, as this is important not only early during infection but also at later stages, when virions are produced. Notably, in contrast to HIV-1, complement-opsonized HIV-1 triggered the RIG-I-like receptors (RLRs) RIG-I and MDA5, leading to MAVS-induced type I IFN responses. Our data strongly suggest that complement receptor 4 (CR4 and CD11c/CD18) present in rafts with CCR5 mediated a very efficient fusion of HIV-1 and increased levels of incoming viral RNA that are more effectively sensed by RLRs. Moreover, HIV-1-mediated suppression of MAVS was prevented via complement receptor signaling. Thus, we have uncovered a novel pathway of HIV-1 sensing via complement receptors and cross talk with CCR5, leading to RLR-dependent type I IFN responses. This pathway might play a role not only in acute-phase responses to HIV-1 but also during the chronic phase, where continuous immune activation is observed.

## RESULTS

### Complement-opsonized HIV-1 induces efficient type I IFN responses by down-modulating Raf-1 and PLK-1 activation.

**FIG 1:**
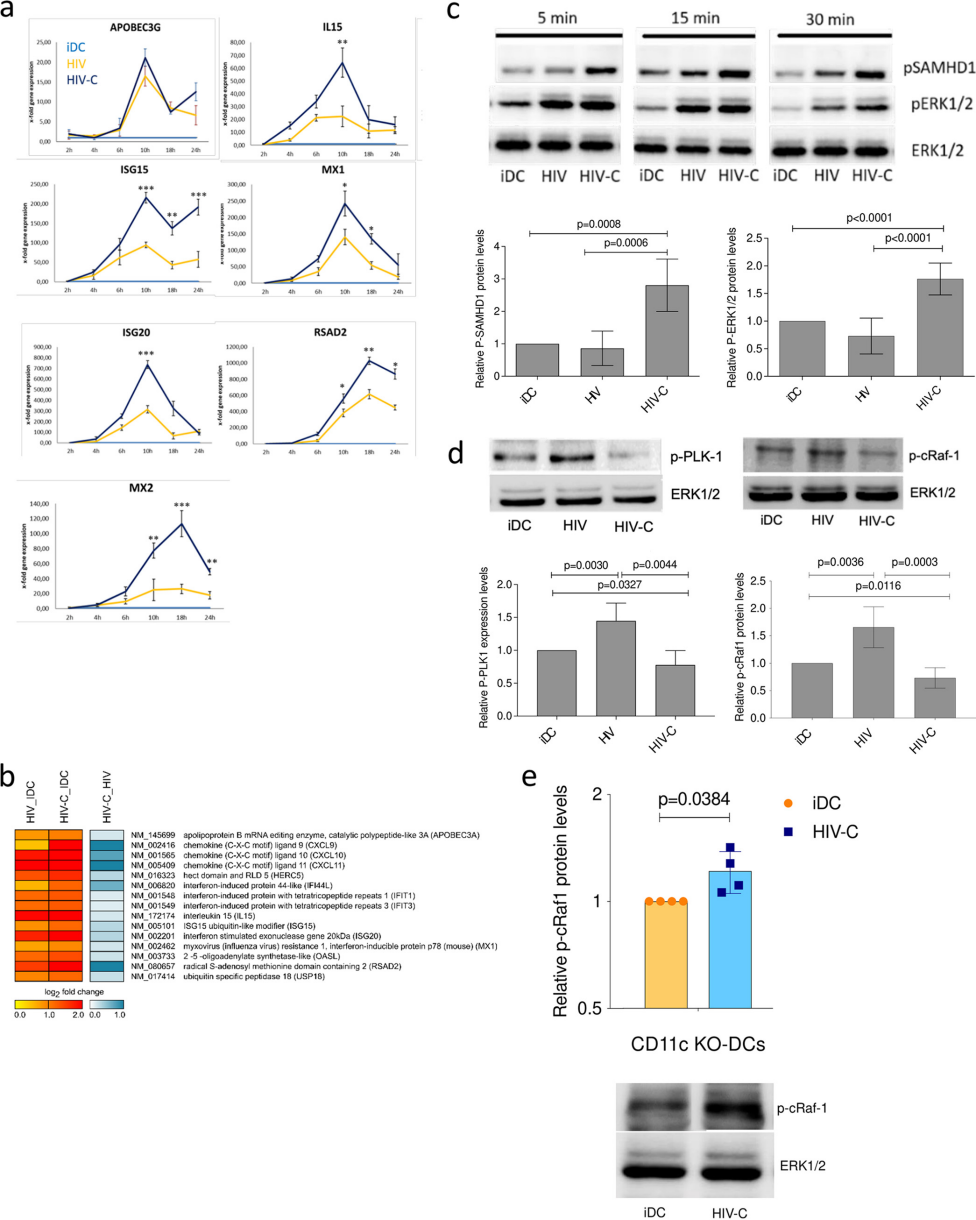
HIV-C initiates an efficient type I IFN response after HIV-1 infection in DCs. (a) Real-time RT-PCR analyses of APOBEC3G, IL-15, MX1, ISG15, ISG20, MX2, and RSAD2 mRNAs in moDCs after infection as indicated with various differentially opsonized (HIV and HIV-C) HIV-1 strains. RT-PCR was performed in duplicate for each sample, and data are means and standard deviations (SD) for cells from three donors exposed to HIV-1 BaL. Unpaired Student's *t* test was performed to analyze statistical significance between HIV and HIV-C. *, *P* < 0.05; **, *P* < 0.01; ***, *P* < 0.001. (b) Microarray analyses of HIV- and HIV-C-exposed DCs after 6 h infection with different strains (BaL, 92BR030, and 92UG037). Cells from four donors exposed to either BaL and 93BR030 or BaL and 92UG037 were analyzed. (c) (Top) Representative immunoblot (IB) analyses of phosphorylated SAMHD1 and ERK1/2 and nonphosphorylated ERK1/2 as a loading control of DCs exposed for short times (5, 15, and 30 min) to differentially opsonized HIV-1 BaL (HIV and HIV-C). IB analyses were repeated in five independent experiments. (Bottom) Quantification of SAMHD1 and ERK1/2 phosphorylation at 4 h after HIV exposure using ImageJ for samples from five donors. (d) (Top) Representative IB analyses of phosphorylated PLK1 and c-Raf-1 (Ser338), and nonphosphorylated ERK1/2 as a loading control of DCs exposed for 4 h to differentially opsonized HIV-1 BaL (HIV and HIV-C). (Bottom) Quantification of PLK-1 and c-Raf-1 phosphorylation at 4 h after HIV exposure using ImageJ for samples from three donors. (e) c-Raf-1 phosphorylation was also analyzed in untreated and HIV-C-exposed CD11c-KO DCs. (Top) Relative c-Raf-1 phosphorylation levels in CD11c-KO DCs from four independent experiments; (bottom) representative IB of p-c-Raf-1 and ERK1/2 as a loading control. Unpaired Student's *t* test was performed to analyze statistical significance between controls or HIV and HIV-C in all analyses.

Here, we investigated the type I IFN responses induced by complement-opsonized HIV-1 (HIV-C) as both monocyte-derived DCs and blood BDCA1^+^ DCs were infected at higher levels with HIV-C (see Fig. S1 in the supplemental material). DC infection was similar to that in HIV-Vpx-loaded DCs by overcoming SAMHD1 restriction (2). The higher infection was further associated with higher DC maturation (Fig. S2) (2). Therefore, we investigated expression of IL-15 and ISGs, such as APOBEC3G, ISG15, ISG20, MX1, MX2, and RSAD2 (Fig. 1a), after infection with R5-tropic, non-opsonized (HIV) and complement-opsonized (HIV-C) HIV-1 strains (BaL and YU-2). Exposure of DCs to HIV-C mediated transient, significantly higher expression of ISGs (ISG15, ISG20, MX1, MX2, and RSAD2) and IL-15 than exposure of DCs to HIV in real-time PCR (Fig. 1a) and/or microarray analyses (Fig. 1b). To assess the higher DC activation and infection by HIV-C than by HIV, the cell signaling intermediates ERK1/2 and SAMHD1 were analyzed (Fig. 1c). In Fig. 1c, the upper panel shows a representative immunoblot of short-term phosphorylation of ERK1/2 and SAMHD1 of control DCs (immature DCs [iDCs]), HIV-exposed DCs, and HIV-1-exposed DCs, while the lower panel shows a summary graph for cells from five donors at the 4-h time point. These analyses revealed that HIV-C induced increased and prolonged phosphorylation of ERK1/2 and SAMHD1 in DCs compared to control DCs (iDCs) and HIV-loaded DCs. Moreover, DCs exposed to HIV-C exhibited lower Raf-1 and PLK1 phosphorylation than noninfected iDCs and HIV-exposed DCs (Fig. 1d). To depict CR4-dependent p-c-Raf-1 reduction, we used CD11c-knockout (KO) THP1 DCs, which were recently characterized by us in detail (22), and analyzed c-Raf-1 phosphorylation in this phenotype. These analyses revealed that following CD11c KO, c-Raf-1 was activated to significantly higher levels than in iDCs (Fig. 1e, top [summary of 4 independent experiments] and bottom [representative immunoblot]). The data illustrated the direct involvement of CR4 in c-Raf-1 repression. Altogether, we found that complement opsonization of HIV initiated an efficient antiviral innate response coupled to higher activation in DCs via a different signaling pathway. Moreover, in HIV-C-exposed DCs, PLK1 and Raf-1 were not activated.

10.1128/mBio.02408-21.1 FIG S1 Higher DC infection with HIV-C in moDCs and BDCA1^+^ DCs. Covalent complement coating of HIV-1 (HIV-C) mediated a significantly higher infection of DCs independent of the subset (left, moDCs; right, BDCA1^+^ DCs) or analysis (left, flow-cytometric analysis of moDCs infected with HIV- or HIV-C-GFP; right, p24 ELISA of supernatants) used. In primary BDCA1^+^ DCs, IB analyses revealed a higher phosphorylation of TBK1 as shown with moDCs (inset, right). Data are means and SD for cells from 3 donors. Download FIG S1, TIF file, 0.1 MB. Copyright © 2021 Posch et al. 2021 Posch et al. https://creativecommons.org/licenses/by/4.0/ This content is distributed under the terms of the Creative Commons Attribution 4.0 International license.

10.1128/mBio.02408-21.2 FIG S2 HIV-C causes higher DC maturation and activation. DCs were exposed for 48 h to LPS (turquoise), HIV (blue), and HIV-C (red) or left untreated (iDC, yellow). LPS- and HIV-C-stimulated DCs showed a highly activated and mature phenotype compared to iDCs and HIV-infected DCs, as measured by fluorescence-activated cell sorting (FACS) analyses using CD83 (upper left), CCR7 (lower left), CD86 (upper right), and CD40 (lower right). FACS analyses were repeated 5 times using DCs from different donors. Download FIG S2, TIF file, 0.3 MB. Copyright © 2021 Posch et al. 2021 Posch et al. https://creativecommons.org/licenses/by/4.0/ This content is distributed under the terms of the Creative Commons Attribution 4.0 International license.

### Infection with complement-opsonized HIV-1 mediates higher MAVS aggregation.

**FIG 2:**
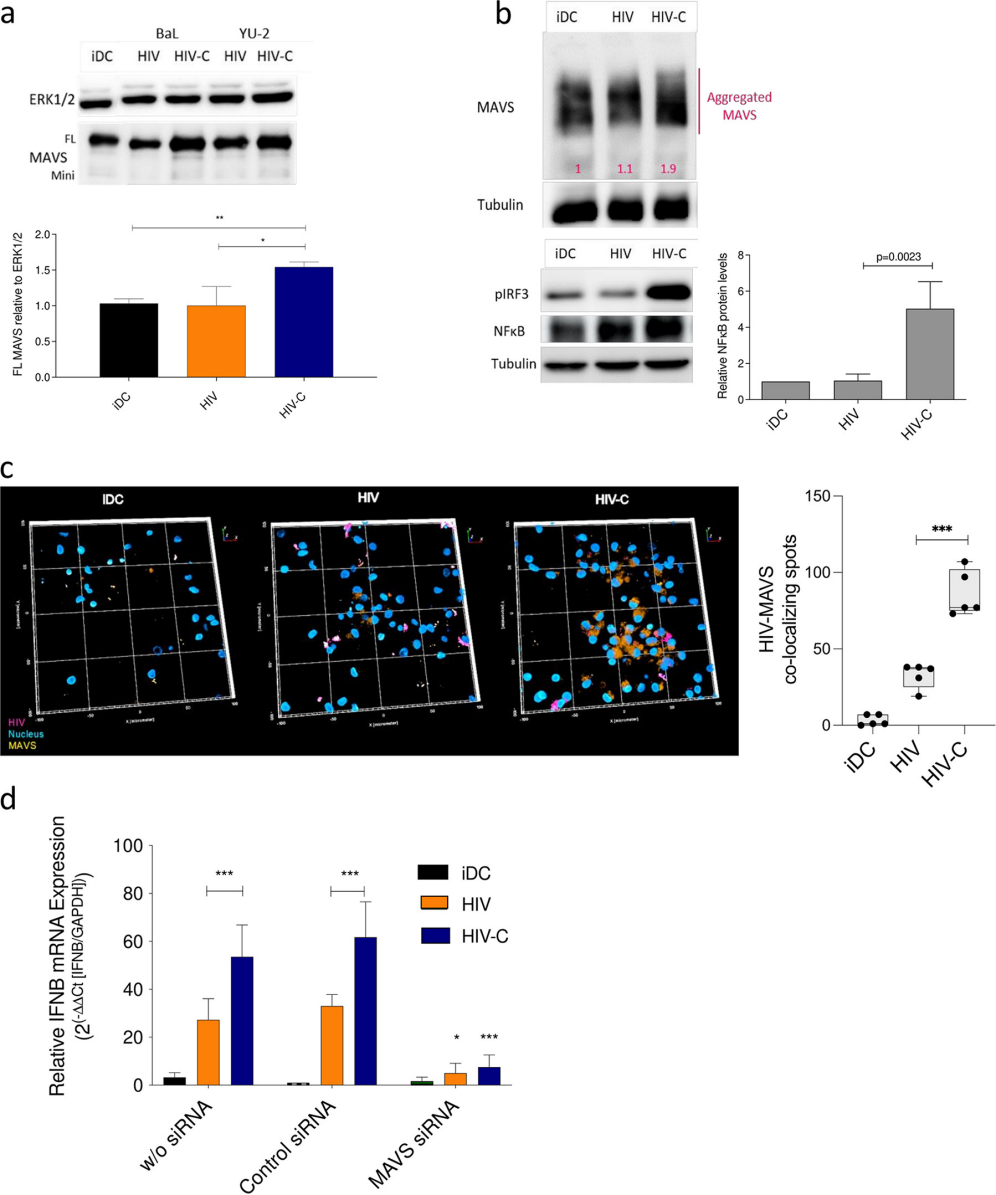
HIV-C mediates efficient type I IFN responses via a MAVS/IRF3/NF-κB signaling axis. (a) Representative IB analyses for one donor of full-length MAVS (FL) and mini-MAVS 9 h after infection of DCs with differentially opsonized HIV-1 (HIV and HIV-C) strains BaL and YU-2. (Top) Representative IB using ERK1/2 as a loading control for IB; (bottom) quantitative results from 3 donors. Statistical significance was analyzed using GraphPad Prism software with one-way ANOVA and Tukeýs posttest. (b) MAVS aggregation was shown under nonreducing conditions in cells from three independent donors using 8% acrylamide native gels in DCs left untreated (iDC) or exposed for 9 h to HIV or HIV-C. IB analyses of phosphorylated IRF3 and activated NF-κB, recognizing the NLS of human p65, are shown. Tubulin was used as a loading control. Quantitative analyses of activated NF-κB from 4 donors are also depicted, and statistical significance was analyzed using unpaired Student's *t* test. (c) (Left) Confocal microscopic analyses of MAVS (orange) and HIV (pink) in iDCs and DCs infected with HIV or HIV-C-mCherry for 9 h. Representative 3D overviews are presented, and the experiment was repeated three times independently. (Right) Spot analyses were performed using RMS spot analysis in the Harmony software (Perkin Elmer), and HIV/MAVS colocalizing spots are illustrated from 5 independent areas and 300 cells in total. Statistical analysis was performed using GraphPad Prism software and unpaired Student's *t* test. (d) RT-PCR analyses of type I IFN (IFN-β) levels after silencing (siRNA) of MAVS expression in moDCs (MAVS siRNA). A control siRNA and moDCs without siRNA served as controls. Data are means and SD for analyses with cells from 4 donors, done in duplicate. A highly significant reduction in IFN-β was observed in DCs treated with MAVS siRNA and infected with HIV-C (red) compared to controls (control siRNA and no siRNA). One-way ANOVA with Tukeýs posttest was performed (*, *P* < 0.05; ***, *P* < 0.001).

Next, we assessed whether type I IFN responses are mediated in HIV-C-exposed DCs via the signaling adaptor MAVS, since HIV-C suppressed Raf-1/PLK1 interactions. The presence of the full-length (FL) MAVS form indicates enhanced aggregation of this signaling adaptor at mitochondrial membranes (23), and MAVS aggregation can be visualized by immunoblotting under reducing conditions. Figure 2a (top) depicts an immunoblot of cells from one donor infected with two different HIV or HIV-C strains (BaL and YU-2), and independent of the virus isolate used, significantly higher FL MAVS was induced in HIV-C-exposed DCs but not iDCs or HIV-exposed DCs (Fig. 2a, top). A summary of data obtained with cells from three donors and two virus isolates is also presented in Fig. 2a (bottom). An ∼2-fold-higher aggregation of MAVS in HIV-C-infected DCs was further seen under nonreducing conditions (Fig. 2b) compared to iDCs (1.0-fold) or HIV-DCs (1.1-fold), and the increased MAVS stimulation resulted in an increased activated downstream IRF3 and NF-κB signaling in HIV-C-DCs but not iDCs or HIV-DCs (Fig. 2b, pIRF3 and NF-κB). Active NF-κB was detected by an NF-κB p65 antibody (Ab) directed against the nuclear localization sequence (NLS) of human p65. Enhanced MAVS/HIV-C aggregation was further seen using confocal microscopic analyses. Confocal imaging highlighted the MAVS/HIV interactome and significantly higher presence and colocalization of MAVS and virus in HIV-C-exposed DCs (Fig. 2c, HIV-C) compared to uninfected iDCs and HIV-exposed DCs (Fig. 2c, iDC and HIV). Silencing MAVS expression by RNA interference (RNAi) abrogated IFN-β expression in HIV-C-infected DCs (Fig. 2d, MAVS siRNA), while scrambled small interfering RNA (siRNA) did not have any impact on type I IFN expression (Fig. 2d, control siRNA). Overall, these results suggest that complement opsonization of HIV-1 mediates aggregation of the signaling adaptor MAVS in DCs, which is associated with IRF3 and NF-κB activation and significantly increased type I IFN responses.

### Infection with HIV-C induces phosphorylation of TBK1.

**FIG 3:**
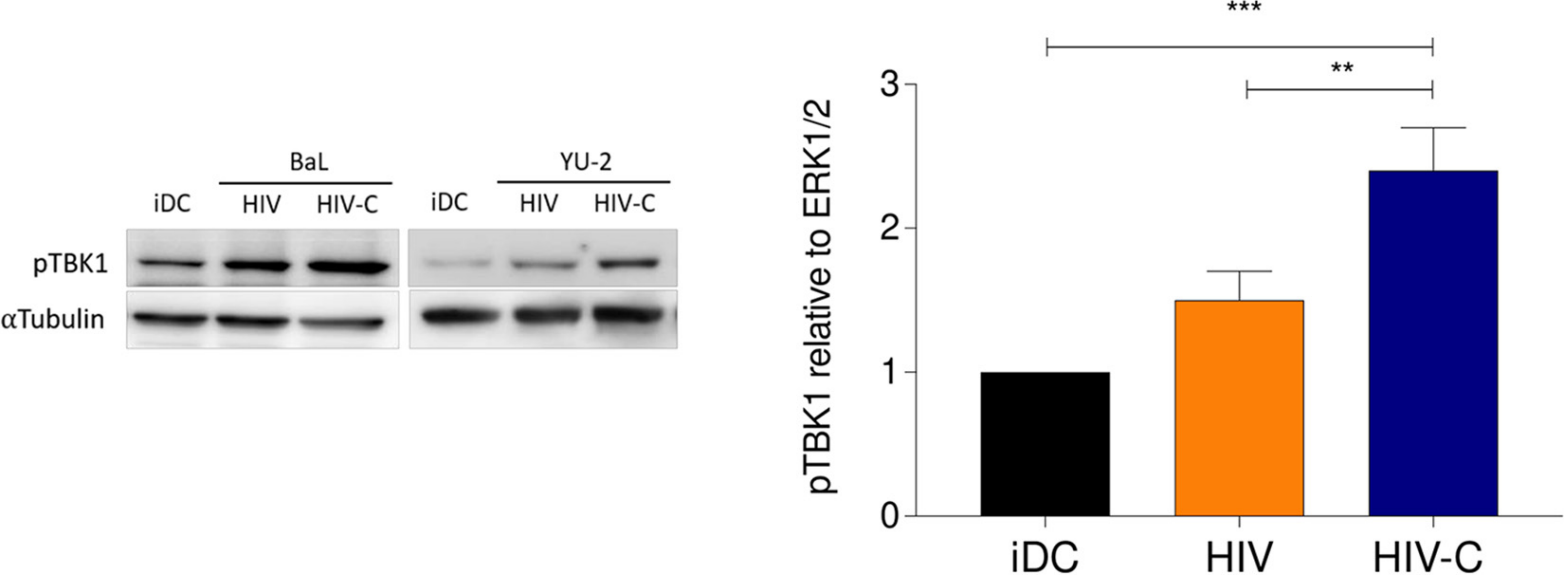
HIV-C mediates efficient type I IFN responses via TBK1 phosphorylation. IB analyses of phosphorylated TBK1 after infection of DCs with differentially opsonized HIV-1 (HIV and HIV-C). (Left) Representative IBs from experiments using BaL or YU-2 for infection with α-tubulin as a loading control for IB; (right) quantitative results from 4 donors infected with BaL and YU-2. One-way ANOVA with Tukey’s posttest was performed (**, *P* < 0.01; ***, *P* < 0.001).

Strikingly, HIV-C-infected DCs efficiently upregulated IRF3 and type I IFN via MAVS. Thus, we next characterized TBK1 activation in monocyte-derived DCs (moDCs) or BDCA-1^+^ DCs exposed to various HIV and HIV-C strains or left untreated (iDC) (Fig. 3 and Fig. S1). In these experiments, we also used BDCA-1^+^ DCs to monitor TBK1 phosphorylation, since, like moDCs, primary BDCA-1^+^ DCs were also infected to significantly higher levels using HIV-C (Fig. S1). Enhanced TBK1 phosphorylation in DCs exposed to HIV was monitored compared to iDCs; however, the differences were not significant (Fig. 3). Interestingly, significantly higher TBK1 phosphorylation was demonstrated in DCs subjected to HIV-C than in iDCs or HIV-exposed DCs, independent of DC and HIV strains (Fig. 3 and Fig. S1, right). Experiments were independently repeated seven times using DCs from different donors. Therefore, in DCs exposed to HIV-C, TBK1 links the MAVS/IRF3/NF-κB axis and acts as a pathway to efficient type I interferon induction by opsonized virus.

### HIV RNA is the motif activating the MAVS/TBK1/IRF3/NF-κB/type I IFN interactome in DCs exposed to complement-opsonized HIV-1.

**FIG 4:**
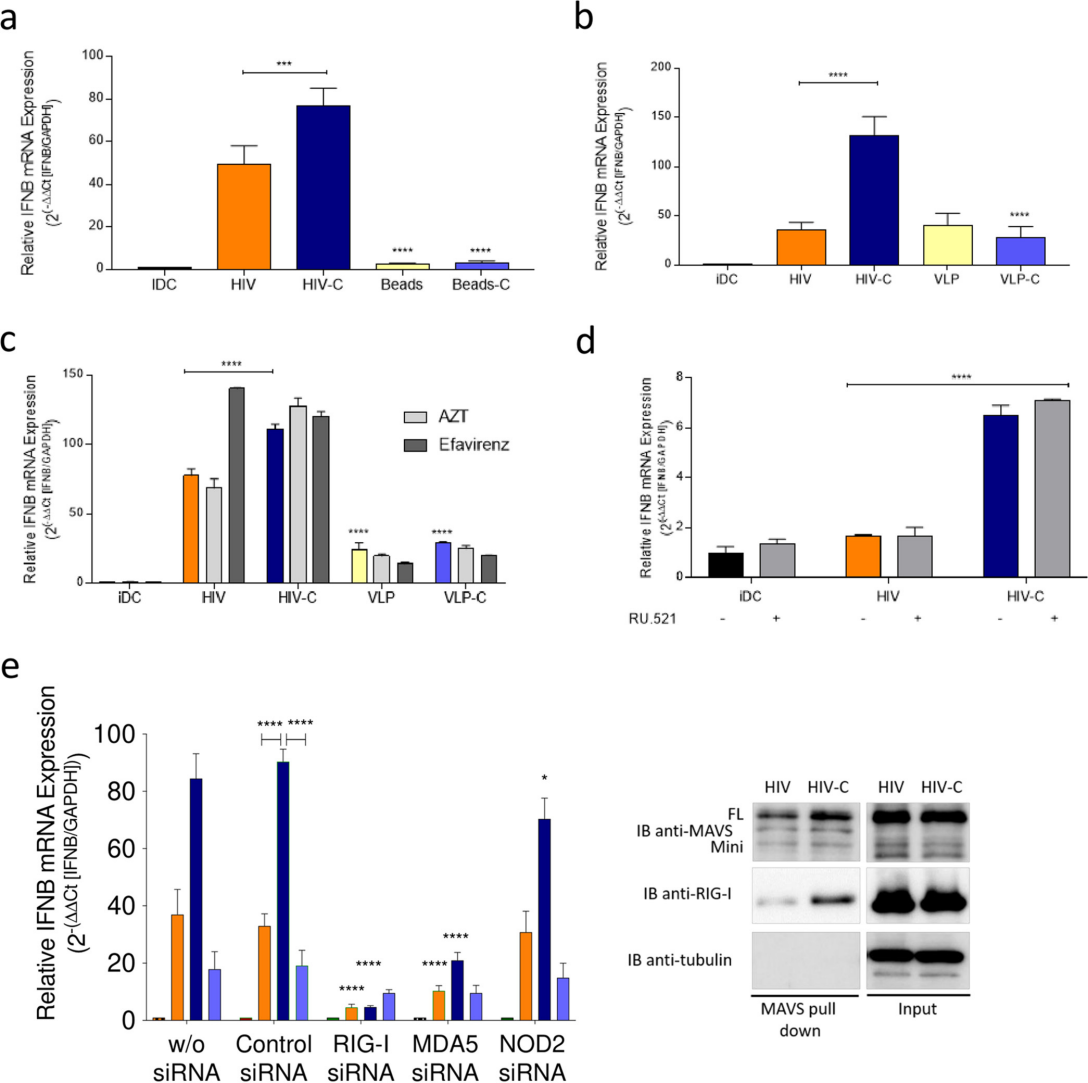
Incoming viral RNA is the PAMP recognized in DCs upon HIV-C infection. (a and b) Real-time RT-PCR analyses of IFN-β mRNA in moDCs after infection with HIV or HIV-C (BaL and YU-2) or differentially opsonized beads of similar size (a) or VLPs (b). Data are means and SD for analyses of cells from four donors, performed in duplicate. One-way ANOVA with Tukeýs posttest was performed (***, *P* < 0.001; ****, *P* < 0.0001) (c) RT-PCR analyses of IFN-β mRNA in moDCs after pretreatment with AZT or efavirenz prior exposure to HIV or HIV-C (BaL and YU-2), VLP, or VLP-C. Data are means and SD for analyses of cells from four donors, performed in duplicate. One-way ANOVA with Tukey’s posttest was performed (****, *P* < 0.0001). (d) RT-PCR analyses of type I IFN (IFN-β) levels after pretreatment with the cGAS inhibitor RU.521 (20 μM) prior exposure to HIV or HIV-C (BaL and YU-2). Data are means and SD for analyses of cells from three donors, performed in duplicate. One-way ANOVA with Tukey’s posttest was performed (****, *P* < 0.0001). (e) RT-PCR analyses of IFN-β levels after silencing RIG-I, MDA-5, or NOD2 expression in moDCs (RIG-I siRNA, MDA5 siRNA, and NOD2 siRNA). A control siRNA and moDCs without siRNA served as controls. Data are means and SD for analyses of cells from 4 donors done in duplicate. A highly significant reduction in IFN-β was observed in DCs treated with MAVS siRNA and infected with HIV-C (red) compared to controls. One-way ANOVA with Tukey’s posttest was performed (*, *P* < 0.05; ****, *P* < 0.0001). IB analyses of FL and mini-MAVS or RIG-I after DC infection with HIV or HIV-C followed by immunoprecipitation using a MAVS Ab directed against another epitope. MAVS pulldown is shown on the left; input is on the right. The pulldown was repeated three times independently.

Next, we characterized in detail the signal triggering the significantly enhanced type I IFN response in HIV-C-infected versus HIV-infected DCs. To determine, whether complement receptor signaling is sufficient to mediate increased type I IFN levels in DCs, cells were exposed to non-opsonized or C-opsonized latex beads (Fig. 4a, beads and beads-C). C-opsonized beads did not mediate higher type I IFN levels than non-opsonized beads. Similar results were observed using non-opsonized or C-opsonized virus-like particles (VLPs) to monitor the impact of the HIV-1-envelope glycoproteins in combination with covalently linked C3 fragments, thereby excluding not only complement receptors or fragments but also HIV envelope as trigger for improved antiviral immunity in HIV-C-infected DCs (Fig. 4b, VLP and VLP-C). The reverse transcription (RT) inhibitors zidovudine (AZT) and efavirenz, which were shown to inhibit viral cDNA (late RT) production *in vitro* (24), did not affect type I IFN induction in HIV-C-infected DCs, suggesting that viral RNA and not DNA is the pathogen-associated molecular pattern (PAMP) triggering the observed enhanced type I IFN expression (Fig. 4c, AZT and efavirenz).

This was further illustrated using the cGAS inhibitor RU.521, which was recently described to potently and selectively block mouse and human cGAS in cell lines and primary cells (25) (Fig. 4d) and siRNAs for the cytoplasmic sensors RIG-I, MDA-5, and NOD2 (Fig. 4e). Inhibiting cGAS did not affect elevated type I IFN expression levels mediated by HIV-C in DCs (Fig. 4d). In contrast, the highly significant increase in IFN-β mRNA expression in HIV-C-DCs was completely abrogated or significantly down-modulated in RIG-I- and MDA5-silenced DCs upon HIV-C treatment (Fig. 4e, left; RIG-I and MDA5 siRNA). Another PRR, NOD2, acting as an intracellular sensor of bacteria (26–28) but also RNA and DNA viruses (29–31) and binding and activating MAVS in response to single-stranded RNA (ssRNA) (31), seemed to play a minor role with respect to induction of elevated type I IFN expression in HIV-C-exposed, NOD2-silenced DCs (Fig. 4e, left; NOD2 siRNA). The HIV-C-mediated RIG-I/MAVS association was further demonstrated by immunoprecipitation of MAVS using whole-cell extracts followed by immunoblotting of RIG-I (Fig. 4e, right). FL MAVS and RIG-I associated with MAVS in DCs exposed to HIV-C- and to lower levels in HIV-infected DCs (Fig. 4e, right; MAVS pulldown). These results imply that HIV-C mediates a high antiviral type I IFN response in DCs via recognition of viral RNA via RLR and MAVS followed by activation of the IκB-kinase related kinase TBK1 and the transcription factor IRF3.

### Complement-opsonized HIV-1 initiates significantly enhanced fusion in DCs in a CR4/CCR5-dependent manner via rafts.

**FIG 5:**
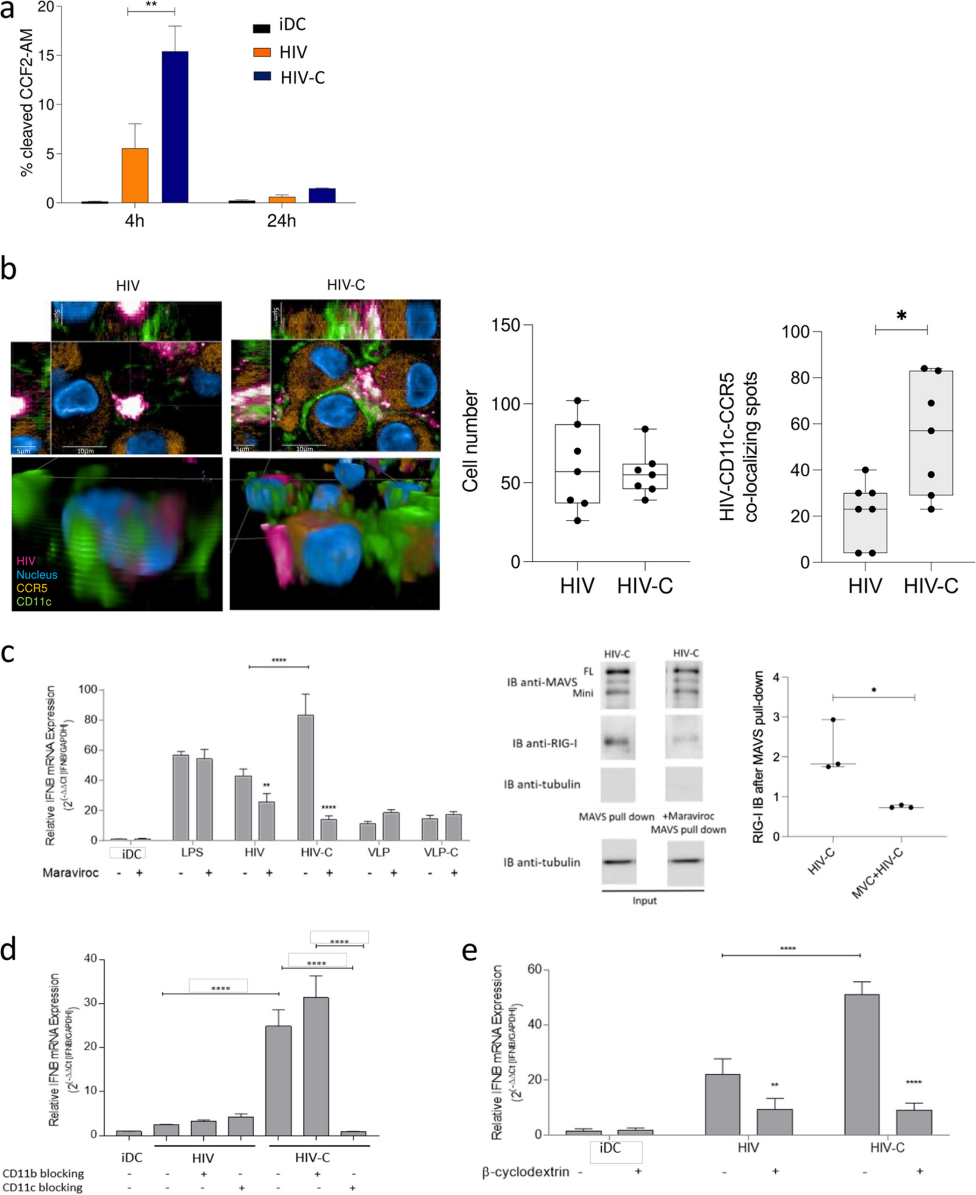
CR4 and CCR5 act in concert via rafts to enhance early type I IFN responses in HIV-C-exposed DCs. (a) BlaM-Vpr analyses of HIV and HIV-C in moDCs after 4 h and 24 h infection. Noninfected DCs served as controls. Data are means and SD from four independent experiments using cells from different donors. Unpaired Student's *t* test was used to characterize differences between HIV and HIV-C (**, *P* < 0.01). (b) (Left) Representative confocal microscopic analyses (top, *xyz* stack; bottom, 3D analysis) of HIV (pink), CD11c (green), CCR5 (orange), and nucleus (blue) in DCs infected 1 h with HIV or HIV-C–mCherry. (Right) Quantitative analyses of DC numbers (white plots) and numbers of HIV-CD11c-CCR5 colocalizing spots (gray plots) are illustrated. For each condition, at least 350 cells were analyzed, and data were generated using the RMS spot analysis of Harmony software (Perkin Elmer). (c) (Left) RT-PCR analyses of type I IFN (IFN-β) levels after blocking CCR5 using maraviroc in LPS-, HIV-, HIV-C, VLP- or VLP-C-exposed moDCs. Data are means and SD for analyses of cells from 4 donors, done in duplicate. In HIV-C-exposed DCs, type I IFN levels were highly significantly down-modulated upon CCR5 blocking (**, *P* < 0.01; ****, *P* < 0.0001), and this too was associated with a reduced RIG-I signal (middle and right) after IP (MAVS pulldown) and IB using MAVS and RIG-I Abs. Tubulin was used as loading control. A representative IB after IP (middle) and the quantification of the RIG-I signal from three independent experiments (right) are depicted. (d) RT-PCR analyses of type I IFN (IFN-β) levels after blocking CR3 using CD11b or CR4 using CD11c in HIV-infected, HIV-C-infected, or noninfected moDCs. Data are means and SD for analyses of cells from 4 donors in duplicate. One-way ANOVA with Tukey’s posttest was performed (****, *P* < 0.0001). (e) RT-PCR analyses of type I IFN (IFN-β) levels after disrupting rafts using β-cyclodextrin in HIV-infected, HIV-C-infected, or noninfected moDCs. Data are means and SD for analyses with cells from 4 donors, done in duplicate. One-way ANOVA with Tukey’s posttest was performed (**, *P* < 0.01; ****, *P* < 0.0001).

After identifying RIG-I and MDA5 as cytoplasmic sensors recognizing viral RNA, we characterized the mechanism of cytoplasmic accumulation of viral RNA in HIV-C-exposed DCs compared to HIV-DCs. For this purpose, we first analyzed the cytoplasmic distribution of HIV or HIV-C in DCs using the Vpr/BlaM assay and found a significantly higher cytoplasmic localization of HIV-C in DCs after 4 h (∼15%) (Fig. 5a). In contrast to this, only low levels of non-opsonized HIV (∼5%) were found in the cytoplasm (Fig. 5a). After 24 h, none of the differentially opsonized virus preparations was located in the cytoplasm (Fig. 5a). To investigate if the higher cytoplasmic distribution and accumulation of HIV-C were associated with CCR5 and CR4, we next performed confocal microscopic analyses using differentially opsonized HIV-mCherry preparations (Fig. 5b, pink). Infected and noninfected cells were stained using CD11c-Alexa Fluor 488 (Fig. 5b, green), CCR5-peridinin chlorophyll green (PerCP)/Cy5.5 (Fig. 5b, orange), and Hoechst (Fig. 5b, blue) as a nuclear stain. DC were exposed to non-opsonized (HIV) and complement-opsonized (HIV-C) HIV together with the CD11c, CCR5, and nuclear stains. After the incubation period, cells were fixed, and images were analyzed using the Operetta HCS and Harmony software-based RMS spot analyses (Perkin Elmer). Image analyses revealed CD11c colocalization with virus and CCR5 in HIV-C-exposed cells (Fig. 5b, HIV-C). In contrast, non-opsonized virus colocalized to significantly lower levels with both CD11c and CCR5 (Fig. 5b, HIV), indicating a correlation with the results from the Vpr-BlaM fusion assay showing significantly more HIV-C than HIV fusion (Fig. 5a). In Fig. 5b, left, *xyz* stacks and a zoomed-in three-dimensional (3D) analysis of single DCs exposed to HIV or HIV-C are shown. Quantitative analyses of DC cell numbers (white plots) and numbers of HIV-CD11c-CCR5 colocalizing spots (gray plots) are illustrated on the right (Fig. 5b). For each condition, at least 350 cells were analyzed and spot analyses were performed.

To characterize the role of CCR5 regarding higher fusion of complement-opsonized HIV-1 in DCs, we performed blocking and immunoprecipitation (IP) experiments using the CCR5 antagonist maraviroc (32) (Fig. 5c). We found that type I IFN induction in HIV-C-exposed DCs was significantly down-modulated upon blocking of CCR5 (Fig. 5c, left). Of course, maraviroc also significantly reduced the already observed low-level expression of type I IFN in HIV-loaded DCs (Fig. 5c, left). VLP-, VLP-C- and lipopolysaccharide (LPS)-exposed DCs and unstimulated DCs were used as controls (Fig. 5c, left). Preincubation of DCs with maraviroc prior to HIV-1 exposure and MAVS immunoprecipitation resulted in significantly lower levels of FL MAVS and RIG-I (Fig. 5c, right). While analysis of whole-cell extracts from HIV-C-exposed DCs again revealed detection of FL MAVS and association of RIG-I with MAVS, this was reduced to basal levels in maraviroc-pretreated HIV-C-DCs (Fig. 5c, right).

To further highlight the importance of CD11c (CR4) with respect to HIV-C-ligation and type I IFN induction in DCs, blocking experiments using CD11b- and CD11c-blocking antibodies were performed. As expected, no changes in type I IFN levels were observed in DCs infected with non-opsonized HIV upon inhibition of CR3 and -4 (Fig. 5d). CD11b blocking on DCs even slightly enhanced the type I IFN expression compared to HIV-C-exposed DCs, while blocking CD11c completely abrogated type I IFN stimulation in HIV-C-DCs (Fig. 5d).

To unravel whether CR4 and CCR5 accumulate in lipid rafts, thereby enhancing cytoplasmic distribution of viral RNA and mediating a significantly improved antiviral capacity of HIV-C-exposed DCs, we disturbed lipid rafts using β-cyclodextrin. These analyses revealed the importance of CCR5/CR4-containing rafts with regard to antiviral immune responses to complement-opsonized HIV-1 from DCs, since disturbing raft formation significantly reduced and nearly abolished type I IFN induction in HIV-C-DCs (Fig. 5e). In addition, the low-level type I IFN induction by HIV was decreased (Fig. 5e). Our data demonstrate that HIV-C aggregates CR4 and CCR5 in lipid raft fractions at the plasma membrane, resulting in significantly higher fusion and shuttling of viral RNA into the cytoplasm of infected DCs associated with significantly enhanced type I IFN induction compared to non-opsonized HIV.

### CR4 depletion is associated with impaired NF-κB activation.

**FIG 6:**
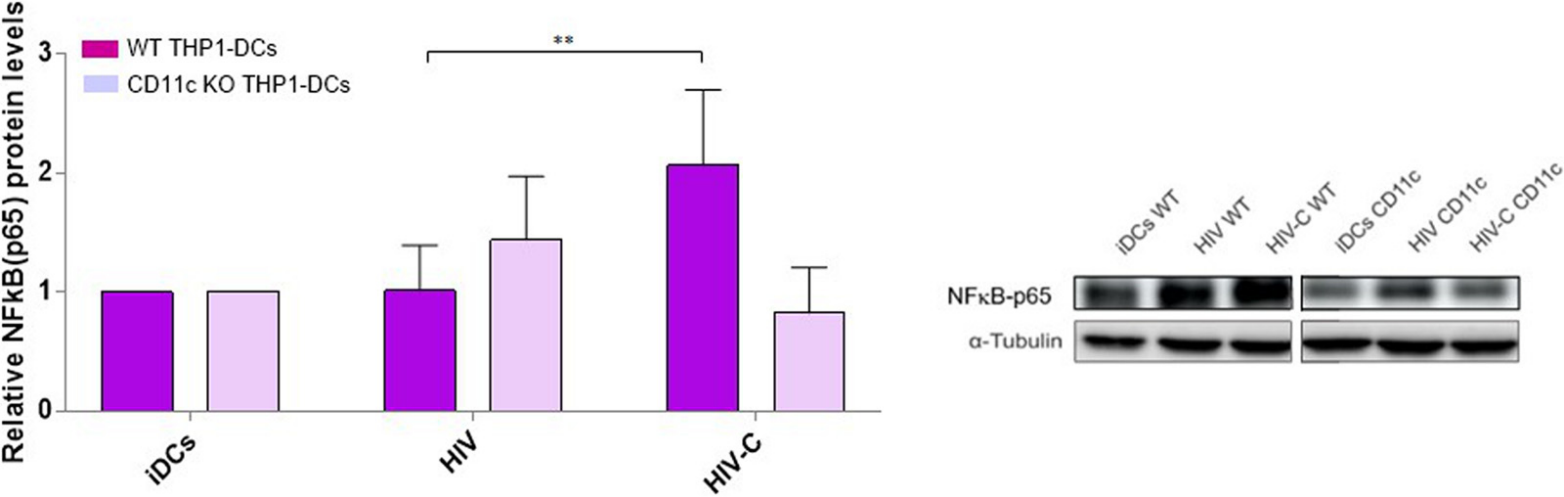
Knocking out CR4 eliminates efficient early type I IFN responses. IB analyses of IRF3 and NF-κB phosphorylation after infection of WT, CD11b-KO, and CD11c-KO THP1 DCs with differentially opsonized HIV-1 (HIV and HIV-C) strains BaL and YU-2. (Left) Quantitative results; (right) representative IB with α-tubulin as a loading control for IB. Data are means and SD for 4 experiments. **, *P* < 0.01.

Since in primary DCs we demonstrated NF-κB activation following HIV-C exposure via IRF3 (2) and an impaired IRF3 phosphorylation in CD11c-KO THP1 DCs (22), we wanted to next unravel whether the described type I IFN-mediating TBK1-IRF3-NF-κB signaling axis (33, 34) is triggered upon CR4 engagement as well. Therefore, we determined activation of NF-κB by using the NF-κB p65-Ab directed against the NLS of human p65. This Ab selectively binds to the activated form of NF-κB. Significantly higher NF-κB activation was detected in wild-type (WT) THP1 DCs loaded with HIV-C, while the NF-κB activation signal was abrogated when CR4 was depleted in CD11c-KO THP1 DCs (Fig. 6). In Fig. 6, the bar chart on the left combines results from 3 independent donors, while a representative immunoblot for NF-κB-p65 is depicted on the right. These data together indicate that the TBK1–IRF3 (22)–NF-κB signaling axis is impaired in CR4-depleted DCs and, as we recently illustrated, is also associated with loss of type I IFN to efficiently signal the viral presence to DCs via CR4 (22).

## DISCUSSION

Not much attention has been paid to the role of complement in the modulation of immune responses to HIV-1; a few studies exist, mainly in mice using ovalbumin (OVA) immunogens, on complement receptor 4 (CR4) targeting to generate efficient T cell responses as well as to promote germinal center induction (35, 36). Using HIV-1, or chemically inactivated HIV-1 for DC exposure, we recently highlighted multiple novel functions of complement receptors, in particular also CR4, for immune modulation and cross talk with other immune effector systems (2, 3, 5, 18, 22, 37). HIV-C was able to overcome SAMHD1 restriction in DCs, which was associated with a significantly improved DC maturation and activation and coupled to induction of highly efficient early type I IFN responses and adaptive anti-HIV-1 immunity compared to the nonprotective immune responses mediated via HIV-exposed DCs (2). Here, we identified CR4 in concert with CCR5 as factors responsible for induction of efficient type I IFN responses in DCs by mediating improved fusion and shuttling of viral particles into the cytoplasm via lipid rafts. Engagement of CR4/CCR5 orchestrated a spontaneous effective antiviral defense via an RLR/MAVS/IRF3/NF-κB/type I interferon axis due to recognition of viral RNA in the cytoplasm.

Type I IFN responses early during infection were illustrated to lower susceptibility to SIV infection in rhesus macaques and to slow disease progression (14), mediating antiviral adaptive immunity by triggering DC maturation (2, 38), CTL induction (39), and T helper polarization (3, 40). These immune responses are effectively induced by complement-opsonized HIV-1 but not non-opsonized HIV-1 (41). Low-level productive DC infection and impaired type I IFN responses due to deficiency in sensing viral DNA or RNA act as evasion strategies by avoiding immune surveillance (16). Gringhuis et al. (21) recently illustrated that the low type I IFN induction in HIV-infected DCs is due to DC-SIGN-dependent activation of Raf-1 and PLK1 that interfered with MAVS aggregation, a prerequisite for downstream IRF3 activation (23). Here, we demonstrated that opsonization of HIV-1 efficiently bypassed PLK1 and Raf-1 activation seen with non-opsonized HIV-1, thereby facilitating early IFN-β production followed by activation of various ISGs and IL-15 in DCs that also exhibit anti-HIV-1 properties (14, 42). Additionally, type I IFNs mediate adaptive immune responses via DC maturation (38), which was displayed when DCs were exposed to HIV-C (2), and DC activation, reflected here by higher SAMHD1 and ERK1/2 phosphorylation mediated by HIV-C. SAMHD1 was described as restricting HIV-1 infection in DCs and myeloid cells by inhibiting reverse transcription due to limiting the deoxynucleoside triphosphate (dNTP) pool and thereby preventing HIV-1 infection of DCs and their activation (43). Other studies demonstrated productive infection of DCs with HIV-1 but lack of maturation and induction of effective antiviral and adaptive immune responses (2, 19, 44).

Productively infected DCs will encounter a plethora of HIV-1 PAMPs (17, 20, 45, 46), such as ssRNA, HIV-1 capsid, DNA, and abortive HIV-1 RNA. We therefore assessed the path taken by complement-opsonized HIV-1, which was associated with highly productive DC infection and efficient type I IFN induction due to overcoming the Raf-1/PLK1 signaling axis. We found a significantly enhanced MAVS aggregation, recruitment of TBK1, and subsequent activation of IRF3 and NF-κB, finally leading to high type I IFN expression in HIV-C- but not HIV-exposed DCs. TBK1, also known as NF-κB-activating kinase, represents one of two noncanonical IκB kinases that is associated with regulation of IRF3 activation and NF-κB signaling pathways (47). Since production of type I IFNs is fundamental in controlling viral infections (48), many viruses have evolved evasion strategies, among these TBK1 modulation, to combat the innate immune mechanisms (49). Binding of HIV-1 to DC-SIGN resulted in PLK1 activation via Raf-1, thereby blocking MAVS aggregation and TBK1 phosphorylation and circumventing a potent type I IFN response (21).

A link between activation of Raf-1 and prevention of dephosphorylation of RIG-I and MDA5 has been reported (50). Our data provide the first evidence that complement coating of viral particles, which is particularly important during HIV-1 transmission and the early stages of acute infection, counteracts HIV-1-mediated evasion from type I IFN induction in DCs by switching on other signaling and uptake mechanisms than non-opsonized HIV-1. HIV-C-mediated type I IFN responses were mostly dependent on the RLR RIG-I, which coimmunoprecipitated with MAVS, and MDA5, thereby identifying incoming RNA as a viral motif. NOD2 did not have any function regarding type I IFN-inducing signals. Viral DNA and envelope were not the PAMPs recognized in early (4-h) HIV-C-infected DCs, as reflected by using complement-opsonized VLPs or blocking interactions using RU.521, AZT, and efavirenz. This is in accordance with work by Elsner et al., who demonstrated that HIV-1 infection does not appear to trigger cGAS-mediated sensing of viral DNA in T cells (51). They concluded that retroviruses, including HIV-1, may have evolved a replication strategy that reduces the abundance of cytoplasmic DNA intermediates to a minimum, thus avoiding susceptibility to cGAS-mediated sensing in infected T cells (51).

Additionally, complement receptor signaling was not sufficient to promote type I IFN expression in HIV-C-exposed DCs, since IFN-β was not initiated in DCs exposed to complement-opsonized beads. The increased amounts of incoming HIV-1 RNA in HIV-C-exposed DCs were illustrated in an enhanced fusion capacity in a CR4- and CCR5-dependent manner via lipid rafts as revealed by blocking experiments using the CCR5 antagonist maraviroc, a CD11c-blocking antibody, and the rafts disruptor β-cyclodextrin. Of course, β-cyclodextrin might interfere not only with signaling events in DCs but also with infection, but at the time analyzed, it is more likely that the raft disruptor hampers antiviral signaling. In contrast to CR4, CR3 was completely dispensable in induction of an efficient antiviral machinery in DCs via complement-opsonized HIV-1. Blocking this receptor via a CD11b-blocking antibody resulted in enhanced type I IFN responses compared to HIV-C-infected DCs.

Enhancement of type I IFN signaling via CR4 was even more prominent in CD11b knockout THP1-DCs, with ∼15-fold higher IFN-β mRNA gene expression, whereas knocking out CD11c resulted in a highly significant, complete abrogation of type I IFN induction, as we recently showed (22). Ellegård et al. (52) described decreased antiviral and inflammatory responses in iDCs exposed to complement-opsonized HIV-1 via CR3, which is in accordance with our results from CD11c-blocking and -depleting experiments (22). In contrast to their data, we detected protective roles of complement-opsonized HIV on primary as well as monocyte-derived DCs, expressing high levels of CR4 besides CR3. Therefore, their observations regarding decreased antiviral effects by exposure of DCs to complement-opsonized HIV-1 might rely on different monocyte isolation or DC differentiation protocols, resulting more in macrophage-like cells with low surface expression of CR4. We illustrated by specific blocking of CD11b here and by CRISPR/Cas9-mediated depletion of CR3 previously (22) that type I IFN responses are significantly augmented, thereby deciphering CR4 as inducer of antiviral immunity in DCs. HIV-1 spontaneously activates and covalently binds complement in semen and at mucosal surfaces and is very well protected against complement-mediated lysis (8, 10, 11). Such complement-opsonized viral particles interact with abundantly expressed CR3 (CD11b and CD18) and CR4 (CD11c and CD18) on DCs, a prominent target of HIV-1 during transmission. Our data underscore the importance of predominantly CR4 in efficiently triggering immediate early antiviral responses, associated with host control and decrease of the reservoir size, as demonstrated in nonhuman primates during SIV infection (14).

Importantly, our study highlights a novel target on DCs, namely, the α chain of CR4, CD11c, for therapeutic interventions in HIV-1 treatment. We found that CD11c targeting and consequently CCR5 accumulation in lipid rafts on DCs mediates a potent antiviral immune response and probably induction of efficient HIV-specific CTL response, as we reported earlier (2, 5). Therefore, targeting CD11c might specifically boost endogenous antiviral immunity, which might be a valuable tool in HIV-1 therapy.

## MATERIALS AND METHODS

### Ethics statement.

Written informed consent was obtained from all participating blood donors by the Central Institute for Blood Transfusion & Immunological Department, Innsbruck, Austria. The use of anonymized leftover specimens for scientific purposes was approved by the Ethics Committee of the Medical University of Innsbruck (EK1166/2018 to D.W.).

### Monocyte isolation and cell lines.

CD14 BD IMAG beads (Becton, Dickinson) were used to isolate monocytes from blood of normal healthy donors according to the manufacturer’s instructions. DCs were generated and analyzed as previously described (2, 37). WT THP1 and KO DCs were generated from the respective THP1 cells by addition of IL-4 (200 U/ml), granulocyte-macrophage colony-stimulating factor (GM-CSF) (300 U/ml), and tumor necrosis factor alpha (TNF-α) (10 ng/ml). 293T cells were cultured in Dulbecco's modified Eagle medium (DMEM) supplemented with 10% fetal bovine serum (FBS).

### Genome editing using CRISPR/Cas9-mediated depletion of CD11b and CD11c and lentiviral transduction.

CRISPR/Cas9-mediated depletion of CD11b and CD11c and lentiviral transduction were performed as recently described (53).

### Plasmids.

Plasmids for HIV-1 (R9-BaL and YU-2) and fluorescently tagged HIV-1 preparations (R9-BaL and pmCherry-Vpr) were obtained from Thomas J. Hope (54, 55). The Vpx expression construct pcDNA3.1Vpx SIVmac239-Myc was used to obtain Vpx-carrying HIV preparations (56).

### Virus production.

Purified HIV stocks were produced as previously described (18). HIV-1 (YU-2 and R9-BaL), HIV-1 mCherry, and Vpx-carrying HIV-1 proviral clones were produced by transfection into HEK293T cells (56). R5-tropic HIV-1 (BaL) was propagated in IL-2–phytohemagglutinin leucoagglutinin (PHA-L)-stimulated peripheral blood mononuclear cells (PBMCs).

### VLP preparation.

Virus-like particles (VLPs) were produced by transfection of HEK293T cells with pMDL-chp6, pSF162 or pADA, and pRSV-Rev at a ratio 6:3:1. Supernatants were harvested at several time points posttransfection, filtered, and concentrated by ultracentrifugation at 20,000 rpm for 90 min at 4°C.

### Opsonization of HIV-1.

To mimic opsonization *in vitro*, purified HIV-1 and VLP stocks were incubated for 1 h at 37°C with human complement (C) serum (Quidel) in a 1:10 dilution as previously described (2). Antibodies used for p24 enzyme-linked immunosorbent assay (ELISA) were kindly provided by Polymun Scientific, Vienna, Austria. A representative virus capture assay (VCA) result is depicted in Fig. S3 and shows deposition of covalently bound C3b/iC3b (C3c Ab) and C3d fragments on HIV-C but not on HIV particles.

10.1128/mBio.02408-21.3 FIG S3 Characterization of the viral surface after opsonization. C3c, C3d, and IgG deposition on the HIV surface opsonized with medium/C3-deficient serum (HIV) or human complement serum (Quidel) (HIV-C) was characterized by VCA assay, as described in Materials and Methods. While HIV did not bind to any of the coated Abs (human C3c, C3d, or IgG), a strong binding of HIV-C to C3c and C3d was observed, and there was only background binding to human IgG. Coating the plate with a mouse IgG Ab served as a negative control for background binding of the virus preparations. The VCA assay is routinely performed after opsonization of HIV, and a representative graph is shown. Download FIG S3, TIF file, 0.08 MB. Copyright © 2021 Posch et al. 2021 Posch et al. https://creativecommons.org/licenses/by/4.0/ This content is distributed under the terms of the Creative Commons Attribution 4.0 International license.

### DC infection.

Day 5 iDCs (1 × 10^5^ cells/100 μl) were infected in triplicate with 25 ng p24/ml differentially opsonized HIV-1 as described elsewhere (18). Briefly, productive infection of DCs and not cell-associated HIV-1 was monitored on several days postinfection by p24 ELISA. Infection was analyzed at least 5 times in different donor cells and using different laboratory and primary HIV-1 isolates, as stated above. Preincubation with inhibitors or blocking antibodies was done for 2 h: 10 μM RU.521 (RU320521; MCE), 5 μg/ml AZT and efavirenz (10 μM; both from the NIH AIDS Research and Reference Reagent Program), 10 μg/ml blocking anti-CD11b (ICRF44) or CD11c (3.9) (BioLegend), maraviroc (2 μM; Sigma-Aldrich), and β-cyclodextrin (5 mM; Sigma-Aldrich). DCs were transfected with 25 nM SMARTgpool siRNA from Dharmacon using the transfection reagent INTERFERin (Polyplus) and used for experiments 48 h later. ON-TARGETplus siRNAs (Dharmacon, Horizondiscovery) used were RIG-I (L-012511-01), MDA5 (L-013041-00), MAVS (L-024237-02), NOD2 (L-003464-01), and nontargeting siRNA (D-001206-13) as a control.

### BlaM-Vpr assay.

Viral fusion was analyzed as described (57). Briefly, the assay relies on incorporation of a β-lactamase Vpr (BlaM-Vpr) protein chimera into the virion, and in target cells, upon fusion, the transfer is monitored by enzymatic cleavage of CCF2, a β-lactamase fluorescent dye substrate. The cleavage causes changes in fluorescence from green (520 nm) to blue (447 nm) that can be monitored by flow cytometry. Only cytoplasmic virions are detected (55). In our experiment, day 5 DCs were plated into a 96-well plate in triplicate (1.5 × 10^5^ cells/100 μl) in RPMI in the presence of 10 mM HEPES (Life Technologies) and 2 mg/ml DEAE-dextran (Sigma-Aldrich). Cells were exposed to the indicated concentrations of non-opsonized (HIV) or complement-opsonized (HIV-C) HIV-1 containing BlaM-Vpr. After 3 h, cells were washed twice in CO_2_-independent medium (Life Technologies), resuspended in CO_2_-independent medium containing 10% fetal calf serum (FCS), and loaded with the CCF2-AM substrate solution (LiveBLAzer FRET-B/G loading kit with CCF2-AM; Life Technologies). After 2 h of incubation at room temperature (dark), cells were washed twice in CO_2_-independent medium and fixed in 4% paraformaldehyde for 30 min, and well contents were pooled for flow-cytometric analysis.

### High-content screening analyses.

To track intracellular HIV localization, iDCs were exposed to mCherry-tagged HIV-1 (250 ng p24/ml). DCs were labeled in addition using Hoechst (nucleus), MAVS-Alexa Fluor 647, CD11c-Alexa Fluor 488, and CCR5-PerCP/Cy5-5 (BioLegend) as indicated in the figures. Confocal microscopy was performed on an Operetta CLS system (Perkin Elmer), and colocalization was analyzed using *xyz* stacks and 3D imaging analyses using the Harmony software (Perkin Elmer). Quantification of cell numbers and spot analyses were done using the RMS Spot Analyses program of the Harmony software (Perkin Elmer). Five to seven fields containing 350 to 500 cells were analyzed, and statistical differences were calculated using unpaired Student's *t* test and GraphPad Prism.

### IB analyses and IP.

For immunoblotting (IB) analyses under reducing (Fig. 2a) or nonreducing (Fig. 2b) conditions, DCs (2 × 10^6^ cells/well) were cultured in starvation medium (RPMI–0.5% FCS) for 3 h and thereafter infected with HIV or HIV-C (500 ng/ml p24) or left untreated and uninfected (iDC). For MAVS, 9 h after infection, cells were lysed and proteins analyzed. IB was done as described by Posch et al. (2). For IP, whole-cell extracts from 2 × 10^6^ HIV-1 infected DCs were used, and IP was performed with 5 μg anti-MAVS (sc-166583; Santa Cruz) used to coat protein G agarose beads (11719394001; Roche). Abs used for IB and IP were phospho-Thr592 SAMHD1 Ab (kindly provided by F. Diaz-Griffero) and SAMHD1, pSAPK/JNK, pERK1/2, pPLK1, pRaf-1, MAVS, pIRF3, NF-κB, TBK1, ERK1/2, and tubulin Abs (all from Cell Signaling Technology). The RIG-I Ab MA5-31715 was from Invitrogen. For every donor, quantification was performed using ImageJ and a loading control to normalize the values (ERK1/2 and tubulin). Quantifications were performed using values from at least 3 donors.

### Relative quantification by real-time RT-PCR.

mRNA expression of IFN-β1, ISGs, and IL-15 was analyzed by real-time RT-PCR as previously described using gene-specific primer-probe pairs (Bio-Rad) (2, 3). All real-time PCR runs were performed on the CFX96 real-time detection system and analyzed using CFX Maestro software (Bio-Rad).

### Microarray analyses.

Microarray analyses were performed as described by Posch et al. (2). Differentially expressed genes were identified using moderated *t* test (R/Bioconductor package limma) in a paired analysis for each DC preparation. *P* values were adjusted for multiple hypothesis testing based on the false discovery rate by the Benjamini-Hochberg method. Genes were considered differentially expressed in one of the treated conditions compared to iDCs if the adjusted *P* value was <0.05 and fold change was ≥2.

### Statistical analysis.

Statistical analysis of differences in gene or protein expression levels was performed utilizing the GraphPad Prism software and dependent on analysis by unpaired Student’s *t* test (two way) or one-way analysis of variance (ANOVA) with Tukey’s posttest. Tests used are indicated in the figure legends.

### Data availability.

All experimental parameters, protocols, and raw and transformed data were submitted to a public repository (ArrayExpress; E-MEXP-3706). Data set information can be accessed via https://www.omicsdi.org/dataset/arrayexpress-repository/E-MEXP-3706. Full data can be accessed via https://www.ebi.ac.uk/arrayexpress/experiments/E-MEXP-3706/.
