# Supplementary figures and images for: Complement Potentiates Immune Sensing of HIV-1 and Early Type I Interferon Responses
Source: mBio. 2021 Oct 12;12(5):e02408-21. doi: 10.1128/mBio.02408-21 (PMC8510548; doi:10.1128/mBio.02408-21)

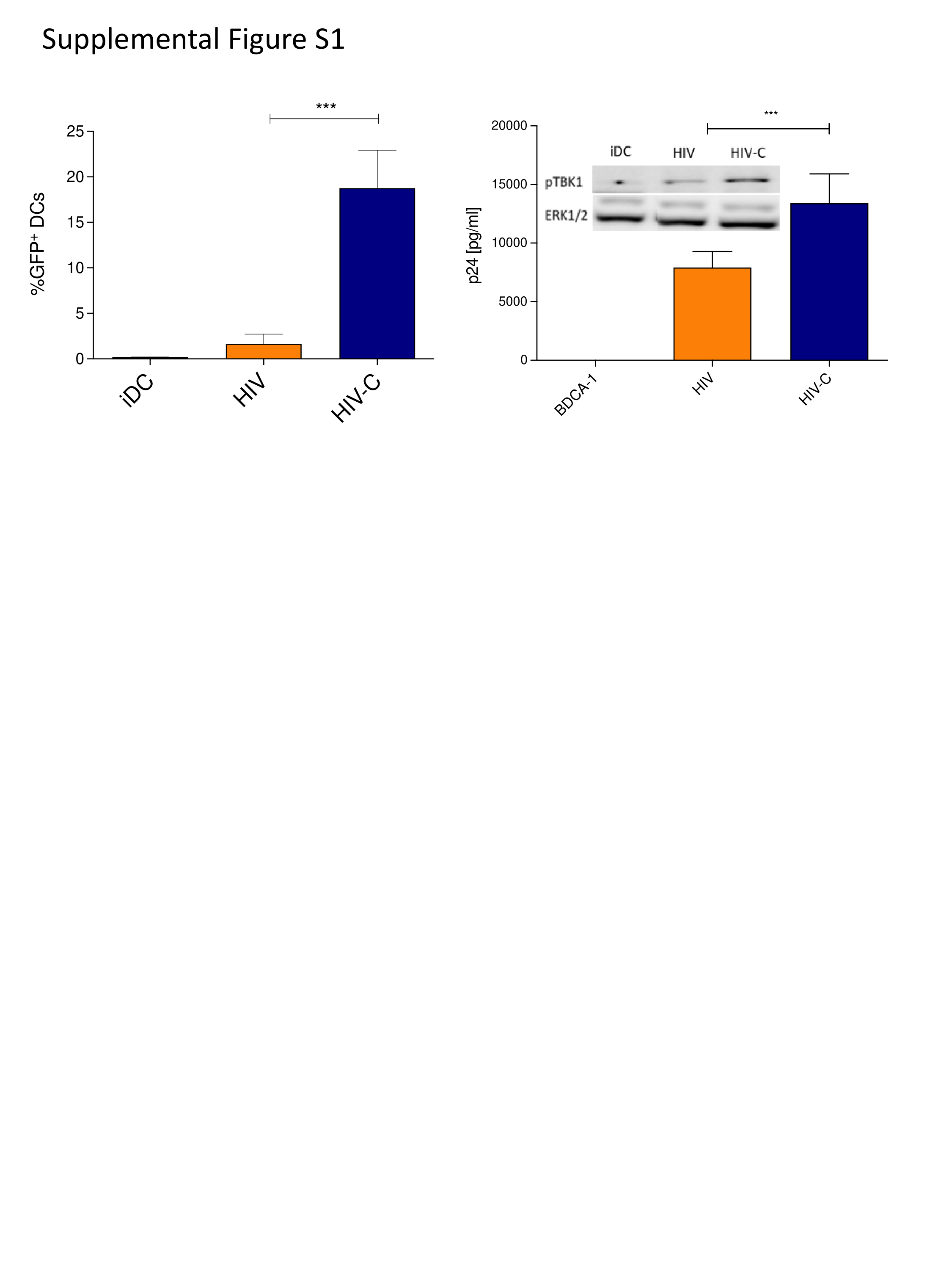

Supplement: FIG S1 [file mbio.02408-21-sf001.tif]

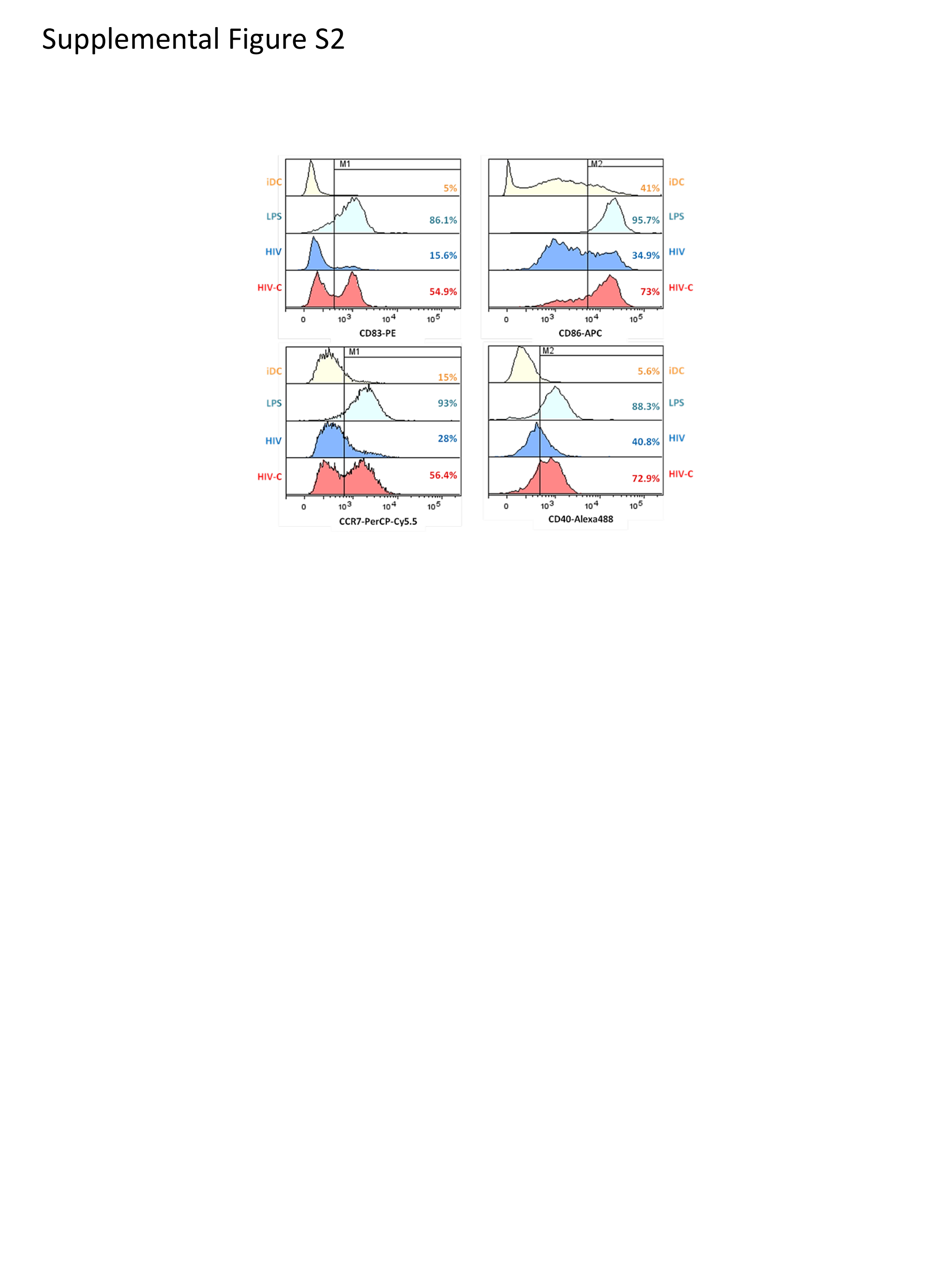

Supplement: FIG S2 [file mbio.02408-21-sf002.tif]

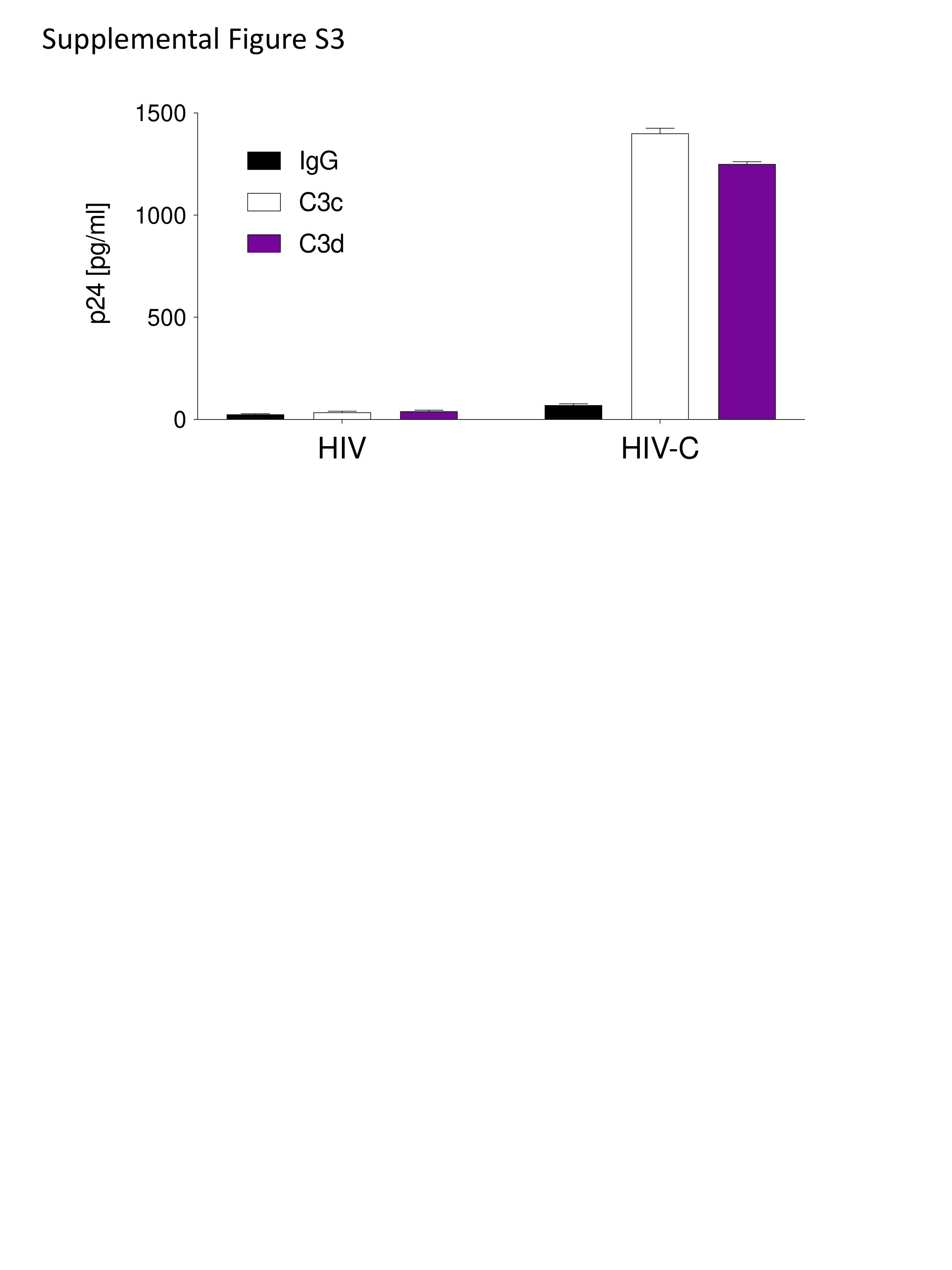

Supplement: FIG S3 [file mbio.02408-21-sf003.tif]
